# Fragility fractures and prescriptions of medications for osteoporosis in patients with polymyalgia rheumatica: results from the PMR Cohort Study

**DOI:** 10.1093/rap/rkab094

**Published:** 2021-11-27

**Authors:** Balamrit Singh Sokhal, Samantha L Hider, Zoe Paskins, Christian D Mallen, Sara Muller

**Affiliations:** 1 School of Medicine, Keele University, Keele; 2 Haywood Academic Rheumatology Centre, Midlands Partnership NHS Foundation Trust, Stoke-on-Trent, Staffordshire, UK

**Keywords:** PMR, osteoporosis, glucocorticoids, cohort, fragility fracture

## Abstract

**Objectives:**

PMR is a common indication for long-term glucocorticoid treatment, leading to an increased risk of osteoporosis and fragility fractures. Guidelines recommend calcium and vitamin D for all patients, in addition to anti-resorptive agents for high-risk patients. The aim of this study was to investigate falls and fragility fracture history and the use of medications for osteoporosis in a PMR cohort.

**Methods:**

Six hundred and fifty-two people with incident PMR responded to a postal survey. Self-reported data on falls, fragility fracture history and medication were collected at baseline. Follow-up data on fragility fractures (hip, wrist and spine) and falls were collected at 12 and 24 months. Logistic regression was used to assess the association between baseline characteristics and fractures.

**Results:**

Fewer than 50% of respondents received osteoporosis treatments, including supplements. One hundred and twelve (17.2%) participants reported a fragility fracture at baseline, 72 participants reported a fracture at 12 months, and 62 reported a fracture at 24 months. Baseline history of falls was most strongly associated with fracture at 12 (odds ratio 2.35; 95% CI: 1.35, 4.12) and 24 months (1.91; 1.05, 3.49) when unadjusted for previous fractures.

**Conclusion:**

Fracture reporting is common in people with PMR. To improve fracture prevention, falls assessment and interventions need to be considered. A history of falls could help to inform prescribing decisions around medications for osteoporosis. Future research should consider both pharmacological and non-pharmacological approaches to reducing fracture risk.

**Key messages**

- Despite guidelines, prescribing of treatments for osteoporosis in patients with PMR remains inadequate.
- Self-reported fragility fractures are common in people with PMR and associated with a history of falls.
- Clinicians should consider falls history when assessing fracture risk and need for medications for osteoporosis.

## Introduction

PMR is a chronic inflammatory condition most prevalent in people >50 years of age, with women being affected more than men [1]. It is characterized by significant bilateral stiffness and muscle pain in the neck, shoulders, hips and thighs, which may be worse in the mornings and after prolonged rest [1]. PMR is often accompanied by an elevated acute phase response [1, 2]. The aetiology of PMR remains unknown [1].

In the UK, the majority of PMR patients are managed exclusively in primary care, with PMR being one of the most common indications for long-term oral glucocorticoid (GC) treatment [3]. Guidelines suggest treatment with an initial GC dose of ≤15 mg for several weeks before gradual reduction, for a regimen commonly lasting ≤2 years, although evidence is emerging that in many cases treatment is continued for longer [2, 4, 5]. Prednisolone is the GC of choice [3]. GC treatment results in increased bone resorption and decreased bone formation, in addition to muscle myopathy, leading to an increased risk of osteoporosis and fragility fractures [6, 7]. Previous work using UK primary care data suggested that people with PMR have a 63% increase in fracture risk (adjusted hazard ratio 1.63; 95% CI: 1.54, 1.73) compared with people without PMR [6]. PMR guidelines recommend calcium and vitamin D supplements for all, with the addition of anti-osteoporosis drugs (e.g. bisphosphonates) to reduce the rate of bone resorption and risk of fragility fractures for all patients aged >65 years and for those with a previous fragility fracture [2]. Osteoporosis guidelines recommend that patients taking GC have a fracture risk assessment, with tools such as the fracture risk assessment tool (FRAX), which includes additional risk factors such as smoking, family history and alcohol, to determine need for bisphosphonates [8]. However, the rates of prescribing of osteoporosis drugs for PMR patients is poor [6, 9]. Falls are known to be associated with fractures and might also contribute to the fragility fractures in patients with PMR; however, the falls history for PMR patients is poorly quantified and might not be addressed [10]. The aim of this study was to examine the frequency of fragility fractures over time and investigate the potential role of falls history and prescribing of therapies for osteoporosis in a cohort of people with recently diagnosed PMR.

## Methods

Full details of study procedures have been reported previously [11]. Briefly, individuals with newly diagnosed PMR were recruited from UK general practice and sent a postal questionnaire [11]. The baseline survey collected information on patient socio-demographics, general health and PMR symptoms [11]. Information on treatment was gathered, including GC prescription, calcium and vitamin D prescription, and anti-osteoporosis prescription, with examples such as bisphosphonates, hormone replacement therapy (HRT) and strontium given [11]. Furthermore, participants reported whether they had fallen in the last 12 months or had fractured their hip, wrist or spine (classified as fragility fractures) [12]. Respondents who consented to follow-up were sent further questionnaires after 1, 4, 8, 12, 18 and 24 months [11]. At months 12 and 24, they were asked to report a history of falls and fragility fractures in the preceding 12 months.

Ethical approval for the PMR Cohort Study was gained from the Staffordshire Local Research Ethics Committee (REC reference number: 12/WM/0021). All participants provided written informed consent.

### Statistical analysis

Simple descriptive statistics were used to describe the sample and the baseline characteristics of those reporting a fracture at 12 and 24 months. Binary logistic regression models were used to assess the association between age, gender, medication use and falls and fragility fractures at 12 and 24 months. Results are presented as odds ratios (OR) with 95% CI. No adjustment was made for previous fractures, because (as expected) they were highly predictive of future fractures [13]. All analyses were performed using IBM SPSS v.26 [14].

## Results

Six hundred and fifty-two (88.2%) of 739 individuals responded to the baseline questionnaire. Four hundred and ninety-six (76.1%) of the 652 responded at 12 months and 446 (68.4%) at 24 months. At baseline, 405 (62.1%) were female, and the mean age was 72.4 (s.d. 9.3) years.

One hundred and twelve (17.2%) respondents reported experiencing a fragility fracture before the baseline survey (Table  1). Of the 112 people reporting a fracture, 8 (7.1%) were aged <60 years, 31 (27.7%) between 60–69 years, 47 (42.0%) between 70–79 years, and 26 (23.2%) were >80 years. At baseline, almost all responders (*n* = 625, 95.9%) had been prescribed prednisolone, and slightly fewer than half were using calcium and vitamin D (304, 46.6%) and a quarter other medication for osteoporosis (170, 26.1%). Of those prescribed medications for osteoporosis, 141 (82.9%) were aged >65 years, and 37 (21.8%) reported previous fractures. One hundred and fifty-one (23.5%) respondents reported a previous fall.

**Table 1:** Self-reported characteristics by fracture status at 12- and 24-month follow-ups [*n* (%)]

Characteristic	Baseline responders (*n* = 652)	Frequency of reported fracture, *n* (%)	
	Baseline	At month 12 (*n* = 72)	At month 24 (*n* = 62)
Fragility fracture before baseline	112 (17.2)	60 (83.3)	49 (79.0)
Female gender	405 (62.1)	50 (69.4)	39 (62.9)
Age, years			
<60	57 (8.70)	5 (6.94)	4 (6.45)
60–69	170 (26.1)	17 (23.6)	16 (25.8)
70–79	274 (42.0)	31 (43.1)	23 (37.1)
≥80	151 (23.2)	19 (26.4)	19 (30.7)
Prescribed prednisolone	625 (97.0)	72 (100)	61 (100)
Prescribed calcium and vitamin D	304 (46.6)	37 (51.4)	29 (46.8)
Prescribed medicine for osteoporosis^a^			
<65 years	29 (4.45)	4 (5.56)	3 (4.84)
≥65 years	141 (21.6)	25 (34.7)	13 (21.0)
Falls	151 (23.5)	27 (37.5)	22 (35.5)
Fragility fracture at 12 months	–	–	45 (72.6)

a Examples given included bisphosphonates, hormone replacement therapy and strontium.

Seventy-two (14.6%) participants reported a fragility fracture at the 12 month survey, and 62 (13.9%) participants reported a fragility fracture at the 24 month survey. Of the 62 who reported a fragility fracture at 24 months, 49 (79.0%) had reported a fragility fracture at baseline and 45 (72.6%) had reported a fragility fracture at 12 months. A history of falls at baseline was significantly associated with fragility fractures at both time points (12 months: 2.35; 1.35–4.12; 24 months: 1.91; 1.05–3.49; Table  2).

**Table 2:** Self-reported characteristics associated with fracture at 12 and 24 months [*n* (%)]

Baseline characteristics	Fragility fracture at month 12	Odds ratio month 12	Fragility fracture at month 24	Odds ratio month 24
	(*n* = 72)	(*n* = 72)	(*n* = 62)	(*n* = 62)
	Unadjusted odds ratio (95% CI)	Adjusted^a^ odds ratio (95% CI)	Unadjusted odds ratio (95% CI)	Adjusted^a^ odds ratio (95% CI)
Female gender	1.59 (0.93, 2.72)	1.41 (0.80, 2.49)	1.24 (0.71, 2.16)	1.03 (0.56, 1.85)
Age, years				
<60	1.00	1.00	1.00	1.00
60–69	1.19 (0.41, 3.45)	1.37 (0.46, 4.08)	1.28 (0.40, 4.12)	1.39 (0.43, 4.55)
70–79	1.22 (0.44, 3.35)	1.30 (0.46, 3.66)	0.94 (0.30, 2.91)	0.90 (0.28, 2.84)
≥80	1.35 (0.47, 3.90)	1.32 (0.45, 3.90)	1.62 (0.51, 5.16)	1.64 (0.51, 5.28)
Prescribed calcium and vitamin D	1.17 (0.71, 1.93)	0.84 (0.47, 1.51)	0.95 (0.56, 1.63)	0.88 (0.48, 1.60)
Prescribed medicine for osteoporosis^b^	2.10 (1.3, 3.48)	2.38 (1.30, 4.35)	0.93 (0.51, 1.72)	0.91 (0.46, 1.82)
History of falls at baseline	2.26 (1.32, 3.85)	2.35 (1.35, 4.12)	1.91 (1.05, 3.49)	1.91 (1.05, 3.49)

a Adjusted for age category, gender, calcium and vitamin D prescription, prescriptions for osteoporosis medicines and falls.

b Example given included bisphosphonates, hormone replacement therapy and strontium.

Prescription of medication for osteoporosis was significantly associated with fragility fracture at 12 months (adjusted OR 2.38; 95% CI: 1.30, 4.35), but not at 24 months (0.91; 0.46–1.82). There was no significant association between age, gender or calcium and vitamin D prescription and subsequent fragility fractures.

## Discussion

People with PMR are at increased risk of osteoporosis because of long-term GC treatment [2]. This study demonstrated that in a cohort of people with incident PMR, fragility fractures and falls were common, with almost one in five reporting a fragility fracture at baseline and almost a quarter reporting a fall in the previous year. In common with the general population, both previous fragility fracture and a history of falls were significantly associated with future fracture [13]. There was an increase in fracture incidence across age categories, although this was not statistically significant.

The guidelines at the time of the baseline survey (2012) suggested that every person with an incident diagnosis of PMR should have been given calcium and vitamin D [2]. However, less than half of the cohort received this treatment. Bisphosphonates were suggested for those at higher fracture risk, for instance being aged ≥65 years or with a prior fragility fracture [2]. Yet in our cohort, only 26% of patients aged ≥65 years reported receiving anti-osteoporosis medication. Although there could be some error around reporting medication (because this was based on self-report with a tick box of medications), this suggests that prescribing of prophylactic treatment for osteoporosis was inadequate relative to PMR and osteoporosis guidelines [2, 13]. Possible reasons for this treatment gap might be related to clinician or patient factors; both clinicians and patients have reported doubts about the need for and safety of bisphosphonates, particularly in the context of multimorbidity [15].

Bisphosphonates were associated with an increased odds of fracture at 12 months. Our study design makes it inappropriate to make any inferences about efficacy; it is likely that this association is explained by bisphosphonates being prescribed to patients with higher fracture risk, thus reflecting confounding by indication. Furthermore, it should be noted that oral bisphosphonates can take several months to take effect on bone density and fracture risk.

In common with previously reported studies, a history of falls at baseline was a significant predictor of future fragility fracture [6]. The incidence of reported falls in respondents aged ≥65 years was 25% at baseline, 22% at month 12 and 27% at month 24. This is concordant with guidelines that suggest people ≥65 years of age have a 30% risk of falls [16]. However, there has been no previous work quantifying the risk of falls in patients with PMR. Given that the causes of falls might be different in this population and might be mediated, in part, by the effects of CSs on muscle mass and the impact of joint pain and stiffness on the risk of falling, it is reassuring to note that the reported risk of falls is broadly in line with that of the general older adult population. Previous studies also suggest that despite guidelines, prescribing for osteoporosis in patients with PMR is inadequate. Our previous work with primary care records suggests that only 13% of people were ever prescribed bisphosphonates. Work using a secondary care PMR cohort of people referred for DXA scan found that 28% of people were prescribed a bisphosphate [6, 17].

Assessment of the risk of falls is not currently included in any UK PMR guidance on fracture prevention, nor is it included in commonly used fracture risk assessment tools, such as FRAX [2, 8]. Clinical decision-making about the appropriateness of fracture prevention treatment needs to incorporate the risk of falls [18]. Furthermore, specific clinical attention needs to be paid to the risk of falls in this population, including to what extent uncontrolled disease or muscle weakness attributable to GC use contributes to this risk. Physiotherapists would be well placed to help manage people at risk of falls or to undertake falls assessment; however, the role of the physiotherapist in PMR is yet to be determined.

A major strength of this study was the recruitment of participants from primary care, ensuring that the study avoided the spectrum bias induced in some studies that recruit exclusively from secondary care [3]. As such, it is likely to be broadly representative of the population diagnosed with and treated for PMR. Another strength was the high response rate at all time points. However, there are some limitations that need to be considered when interpreting the results. This was a questionnaire study and, as such, both fractures and medication were self-reported. Recall bias might have affected our results. Our reported fracture rate was higher than the rate identified in a previous study using health-care record data. The month 12 and 24 questionnaires asked whether patients had experienced a fracture of the hip, wrist or spine in the past 12 months [11]. Participants might have reported a single fragility fracture in duplicate at both 12 and 24 months owing to telescoping [19]. Previous evidence suggests that self-reported fractures can contain ∼14% false positives [20].

However, that was probably counterbalanced by under-reporting of vertebral fractures; spinal fractures that were asymptomatic or undiagnosed will not have been reported and, as such, are likely to represent an underestimate [21]. Additionally, we do not know how many people underwent a bone mineral density scan or had a FRAX assessment and therefore had a formal diagnosis of osteoporosis or were classified as high fracture risk.

For the baseline questionnaire, a time frame for previous fracture was not given [11]. This means that any fracture in those areas at any time in the participant’s life could have been included, with these answers subject to the participant’s ability to recall their fracture history accurately.

Questions regarding medications for osteoporosis were taken from the baseline survey and were self-reported [11]. As such, we were unable to ascertain whether changes to treatment over time had an impact on the likelihood of fracture. It is also possible that although examples of medications were given in the question, prescriptions might have been under-reported, particularly if they related to medications that were not given as examples. However, we gave the most frequently prescribed examples at the time of the survey [11].

CSs increase the risk of osteoporosis, with higher doses increasing this risk further [6]. For this survey, CS dose was self-reported. Not all patients who responded to the survey were able to comment accurately on their CS dose. Therefore, data on CS dose were insufficiently complete or reliable to be included within the analysis.

Sensitivity analyses including previous history of fragility fractures in the adjusted models were conducted. The risk of future fracture in the presence of a previous fracture is well documented as being double compared with groups who have not experienced a prior fracture [8]. This was reflected in our data. Given that this is non-modifiable, we sought to investigate other factors that could be associated with fracture and therefore excluded previous fragility fractures from adjusted analyses.

In summary, this study suggests that despite the well-recognized risk of osteoporosis and fragility fractures with long-term GC use, the prescribing of medications to prevent osteoporosis remains inadequate. Further work is needed to understand fully why bone protection is not prescribed more frequently in this at-risk group and support increased prescribing. A history of falls was also a significant predictor for future fracture. The findings are an important reminder to clinicians to ask about falls history in patients with PMR and consider interventions, such as physiotherapy, which might be of benefit, although there remains an evidence gap for non-pharmacological treatments for PMR. Given the impact of osteoporotic fractures on patients and health services, further studies to improve management are needed urgently to address these unmet needs.
